# The COX-2-derived PGE_2_ autocrine contributes to bradykinin-induced matrix metalloproteinase-9 expression and astrocytic migration via STAT3 signaling

**DOI:** 10.1186/s12964-020-00680-0

**Published:** 2020-11-23

**Authors:** Tsong-Hai Lee, Pei-Shan Liu, Ming-Ming Tsai, Jiun-Liang Chen, Su-Jane Wang, Hsi-Lung Hsieh

**Affiliations:** 1 Stroke Center and Stroke Section, Department of Neurology, Chang Gung Memorial Hospital, Linkou Medical Center, College of Medicine, Chang Gung University, Taoyuan, Taiwan; 2 Department of Microbiology, Soochow University, Taipei, Taiwan; 3 Department of Nursing, Division of Basic Medical Sciences, Research Center for Chinese Herbal Medicine, Graduate Institute of Health Industry Technology, Chang Gung University of Science and Technology, 261 Wenhua 1st Road, Guishan, Taoyuan Taiwan; 4 Department of General Surgery, Chang Gung Memorial Hospital, Chiayi, Taiwan; 5 Division of Chinese Internal Medicine, Center for Traditional Chinese Medicine, Chang Gung Memorial Hospital, School of Traditional Chinese Medicine, College of Medicine, Chang Gung University, Taoyuan, Taiwan; 6 School of Medicine, Fu Jen Catholic University, New Taipei City, Taiwan; 7 Department of Neurology, Chang Gung Memorial Hospital, Taoyuan, Taiwan

**Keywords:** Bradykinin, Matrix metalloproteinase-9, COX-2/PGE_2_ autocrine, STAT3, Brain astrocytes, Neuroinflammation

## Abstract

**Background:**

The matrix metalloproteinase-9 (MMP-9) is up-regulated by several proinflammatory mediators in the central nervous system (CNS) diseases. Increasing reports show that MMP-9 expression is an inflammatory biomarker of several CNS disorders, including the CNS inflammation and neurodegeneration. Bradykinin (BK) is a common proinflammatory mediator and elevated in several brain injury and inflammatory disorders. The raised BK may be detrimental effects on the CNS that may aggravate brain inflammation through MMP-9 up-regulation or cyclooxygenase-2 (COX-2)-derived prostaglandin E_2_ (PGE_2_) production in brain astrocytes. However, the relationship between BK-induced MMP-9 expression and COX-2-derived PGE_2_ release in brain astrocytes remains unclear.

**Methods:**

Herein we used rat brain astrocytes (RBA) to investigate the role of the COX-2/PGE_2_ system in BK-induced MMP-9 expression. We used zymographic, RT-PCR, EIA, and Western blotting analyses to confirm that BK induces MMP-9 expression via a COX-2/PGE_2_-dependent pathway.

**Results:**

Our results show activation of native COX-2 by BK led to PGE_2_ production and release. Subsequently, PGE_2_ induced MMP-9 expression via PGE_2_ receptor (EP)-mediated c-Src, Jak2, ERK1/2, and then activated signal transducer and activator of transcription 3 (STAT3) signaling pathway. Finally, up-regulation of MMP-9 by BK via the pathway may promote astrocytic migration.

**Conclusion:**

These results demonstrated that a novel autocrine pathway for BK-induced MMP-9 protein expression is mediated through activation of STAT3 by native COX-2/PGE_2_-mediated c-Src/Jak2/ERK cascades in brain astrocytes.

**Video Abstract**

## Background

The cyclooxygenase-2 (COX-2), known as prostaglandin (PG)-endoperoxide synthase, is inducible expressed in several tissues by various stimuli to promote PGs biosynthesis, PGE_2_ especially, during inflammatory responses in several cell types [1–4]. Previous studies have shown that overexpression of COX-2 is detected in various inflammatory tissues including macrophages and vascular cells of patients with atherosclerosis. Several evidences have further indicated COX-2 as a major therapeutic target for the treatment of inflammatory disorders [1]. Moreover, homozygous deletion of the COX-2 gene in mice leads to a striking reduction of endotoxin-induced inflammation [5]. Therefore, COX-2 may play a crucial role in the development of various inflammatory disorders. In brain, up-regulation of COX-2 leads to increased production of PGs which may be associated with the central nervous system (CNS) inflammation and neurodegenerative disorders [6]. Moreover, we have demonstrated that several proinflammatory mediators like bradykinin (BK) can induce COX-2 expression and PGE_2_ production in brain astrocytes [7]. Thus, the COX-2/PGE_2_ system may exert as a critical pathological mediator in brain inflammatory diseases.

Matrix metalloproteinases (MMPs) are a large family of zinc-dependent endopeptidases which is a crucial molecule for the turnover of extracellular matrix (ECM) and pathophysiological processes [8]. In the CNS, MMPs, MMP-9 especially, has been demonstrated to participate in morphogenesis, wounding healing, and neurite outgrowth [9]. Several lines of evidence have showed that up-regulation of MMP-9 may contribute to the pathogenic process of brain diseases by several brain injuries [10]. Moreover, several proinflammatory mediators such as cytokines and endotoxin have been shown to induce MMP-9 expression and activity in rat brain astrocytes [11, 12]. Our previous studies have showed that several proinflammatory mediators including BK can induce MMP-9 expression and MMP-9-related functions in brain astrocytes [13]. These studies indicated that MMP-9 may play a critical role in brain inflammation and disorders, and this has aroused our interest to investigate the correlation of COX-2/PGE_2_ system with MMP-9 regulation in brain astrocytes. Here, we used the model in RBA cells to investigate the role of COX-2/PGE_2_ system in BK-induced MMP-9 expression and the relative events like cell migration.

The astrocytes are one type of glial cells in the CNS, which have been proposed to exert a wide range of functions including participating in the immune and repairing responses to brain injury and diseases [14, 15]. Following injury to the human CNS, astrocytes become reactive and respond in stereotypical manner termed astrogliosis [16] which is characterized by astrocyte proliferation and functional changes in inflammatory diseases [17]. In brain, BK and related peptides are released during trauma, stroke, and neurogenic inflammation [18–20], which may play a critical role in the initiation of the CNS inflammatory diseases. All these pathophysiological processes may be involved in inflammatory reactions which were regulated by COX-2/PGE_2_ system. However, the effect of COX-2/PGE_2_ system on BK-induced MMP-9 expression are still unclear, although we have demonstrated that BK induces COX-2 and MMP-9 expression in brain astrocytes [7, 21].

Astrocytes are known to express B2 BK receptor [15, 22], a heterotrimeric G protein-coupled receptor (GPCR) that has been thought to be coupled to PLCβ via interaction with Gq proteins [23]. Activation of BK receptors may induce cell response or gene expression via several signaling molecules, including PKCs, Ca^2+^, and mitogen-activated protein kinases (MAPKs) in several cell types [24–26]. In addition, BK has been shown to regulate the activity and expression of COX-2 through different mechanism in diverse cell types including astrocytes [7, 27, 28]. Likewise, BK induces the activity and expression of MMP-9 via several pathways in brain astrocytes [21, 29]. However, the signaling mechanisms underlying BK-stimulated COX-2-derived PGE_2_ release associated with MMP-9 gene expression in brain astrocytes remain unclear. Thus, the involvement of COX-2/PGE_2_ system in the up-regulation of MMP-9 expression by BK was also under research.

In this study, we investigated the molecular mechanisms underlying BK-induced MMP-9 expression in rat brain astrocytes (RBA). These results suggested that BK-induced MMP-9 expression is mediated through activation of COX-2-derived PGE_2_ release. The released PGE_2_ acts as autocrine signals to activate c-Src, Jak2, ERK1/2, and STAT3 via PGE_2_ receptor (EP)-dependent manner leading to up-regulation of MMP-9 in RBA cells. These results provide new insights into the inflammatory mechanisms of BK and COX-2/PGE_2_ action which may be recognized as therapeutic targets in brain inflammatory diseases.

## Materials and methods

### Materials

Dulbecco’s modified Eagle’s medium (DMEM)/F-12 medium, fetal bovine serum (FBS), and TRIzol were from Invitrogen (Carlsbad, CA). Hybond C membrane and enhanced chemiluminescence (ECL) Western blot detection system were from GE Healthcare Biosciences (Buckinghamshire, UK). Phospho-c-Src (Cat# 6943), phospho-Jak2 (Cat# 3776), phospho-ERK1/2 (Cat# 4370), phospho-STAT3 (Cat# 9145), COX-2 antibody (Cat# 12,282) antibodies were from Cell Signaling (Danver, MA). Anti-glyceraldehyde-3-phosphate dehydrogenase (GAPDH, Cat# GTX627408) antibody was from GeneTex (Irvine, CA, USA). Celecoxib (CLC), AG490, U0126, cucurbitacin E (CBE), Sc-19220, L798-106, and GW627368 were from Santa Cruz (Santa Cruz, CA). PP1 was from Biomol (Plymouth Meeting, PA). Bicinchoninic acid (BCA) protein assay reagent was from Pierce (Rockford, IL). Bradykinin (BK), enzymes, and other chemicals were from Sigma (St. Louis, MO).

### Cell cultures and treatments

The rat brain astrocytic cell line (RBA, CTX TNA2) was purchased from BCRC (Hsinchu, Taiwan) and used throughout this study. Cells were plated onto 12-well culture plates and made quiescent at confluence by incubation in serum-free DMEM/F-12 for 24 h, and then incubated with BK at 37 °C for the indicated time intervals. When the inhibitors were used, cells were pretreated with the inhibitor for 1 h before exposure to BK. Treatment of RBA with these inhibitors alone had no significant effect on cell viability determined by an XTT assay (data not shown).

### MMP gelatin zymography

Growth-arrested cells were incubated with BK for the indicated time intervals. After treatment, the cultured media were collected and analyzed by gelatin zymography [22]. Gelatinolytic activity was manifested as horizontal white bands on a blue background. Because cleaved MMPs were not reliably detectable, only pro-form zymogens were quantified.

### Total RNA extraction and real time-PCR analysis

Total RNA was extracted from RBA cells [22]. The cDNA obtained from 0.5 μg total RNA was used as a template for PCR amplification. Oligonucleotide primers were designed on the basis of Genbank entries for rat MMP-9 and GAPDH. The primers were:

$$\begin{aligned} {\text{MMP-9}}: & {5}\prime {\text{-AGTTTGGTGTCGCGGAGCAC-3}}\prime \;({\text{sense}}) \\ & \;{5}\prime {\text{-TACATGAGCGCTTCCGGCAC-3}}\prime \;({\text{antisense}}) \\ \end{aligned}$$

$$\begin{aligned}\upbeta {\text{-actin}}: & {5}\prime {\text{-GAACCCTAAGGCCAACCGTG-3}}\prime \;({\text{sense}}) \\ & \;{5}\prime {\text{-TGGCATAGAGGTCTTTACGG-3}}\prime \;({\text{anti-sense}}) \\ \end{aligned}$$

The amplification was performed in 30 cycles at 55 °C, 30 s; 72, 1 min; 94 °C, 30 s. PCR fragments were analyzed on 2% agarose 1X TAE gel containing ethidium bromide and their size was compared with a molecular weight markers. Amplification of β-actin, a relatively invariant internal reference RNA, was performed in parallel, and cDNA amounts were standardized to equivalent β-actin mRNA levels.

### Preparation of cell extracts and Western blot analysis

Growth-arrested cells were incubated with BK at 37 °C for the indicated time intervals. The cells were washed with ice-cold phosphate-buffered saline (PBS), scraped, and collected by centrifugation at 45,000×*g* for 1 h at 4 °C to yield the whole cell extract, as previously described [21]. Samples were analyzed by Western blot, transferred to nitrocellulose membrane, and then incubated overnight using an anti-phospho-c-Src, phospho-Jak2, phospho-ERK1/2, phospho-STAT3, or GAPDH antibody. Membranes were washed four times with TTBS for 5 min each, incubated with a 1:2000 dilution of anti-rabbit horseradish peroxidase antibody for 1 h. The immunoreactive bands were detected by ECL reagents and captured by a UVP BioSpectrum 500 Imaging System (Upland, CA). The image densitometry analysis was quantified by an UN-SCAN-IT gel software (Orem, UT).

### Measurement of PGE_2_ release

The cells were seeded in 12-well plates and grew to confluence. Cells were shifted to serum-free DMEM/F-12 medium for 24 h, and then incubated with BK for various time intervals. The culture supernatants were collected to measure PGE_2_ levels using an EIA kit as specified by the manufacturer (Cayman Chemical).

### Transient transfection with siRNAs

Transient transfection of small interfering RNA (siRNA) duplexes corresponding to rat COX-2 and scrambled siRNAs (100 nM) was performed using a Lipofetamine™ RNAiMAX reagent (Invitrogen) according to the manufacturer’s instructions.

### Cell migration assay

RBA cells were cultured to confluence in 6-well culture plates and starved with serum-free DMEM/F-12 medium for 24 h. The monolayer cells were manually scratched with a pipette blue tip to create extended and definite scratches in the center of the dishes with a bright and clear field (~ 2 mm). The detached cells were removed by washing the cells once with PBS. Serum-free DMEM/F-12 medium with or without BK was added to each dish as indicated after pretreatment with the inhibitors for 1 h, containing a DNA synthesis inhibitor hydroxyurea (10 μM) during the period of experiment [29]. Numbers of migratory cells were counted from the resulting four phase images for each point and then averaged for each experimental condition. The data presented are summarized from three separate assays.

### Statistical analysis of data

All data were estimated using GraphPad Prism Program (GraphPad, San Diego, CA). Quantitative data were analyzed by one-way ANOVA followed by Tukey’s honestly significant difference tests between individual groups. Data were expressed as mean ± SEM. A value of *P* < 0.05 was considered significant.

## Results

### Effect of celecoxib on BK-induced MMP-9 expression in brain astrocytes

**Fig. 1:**
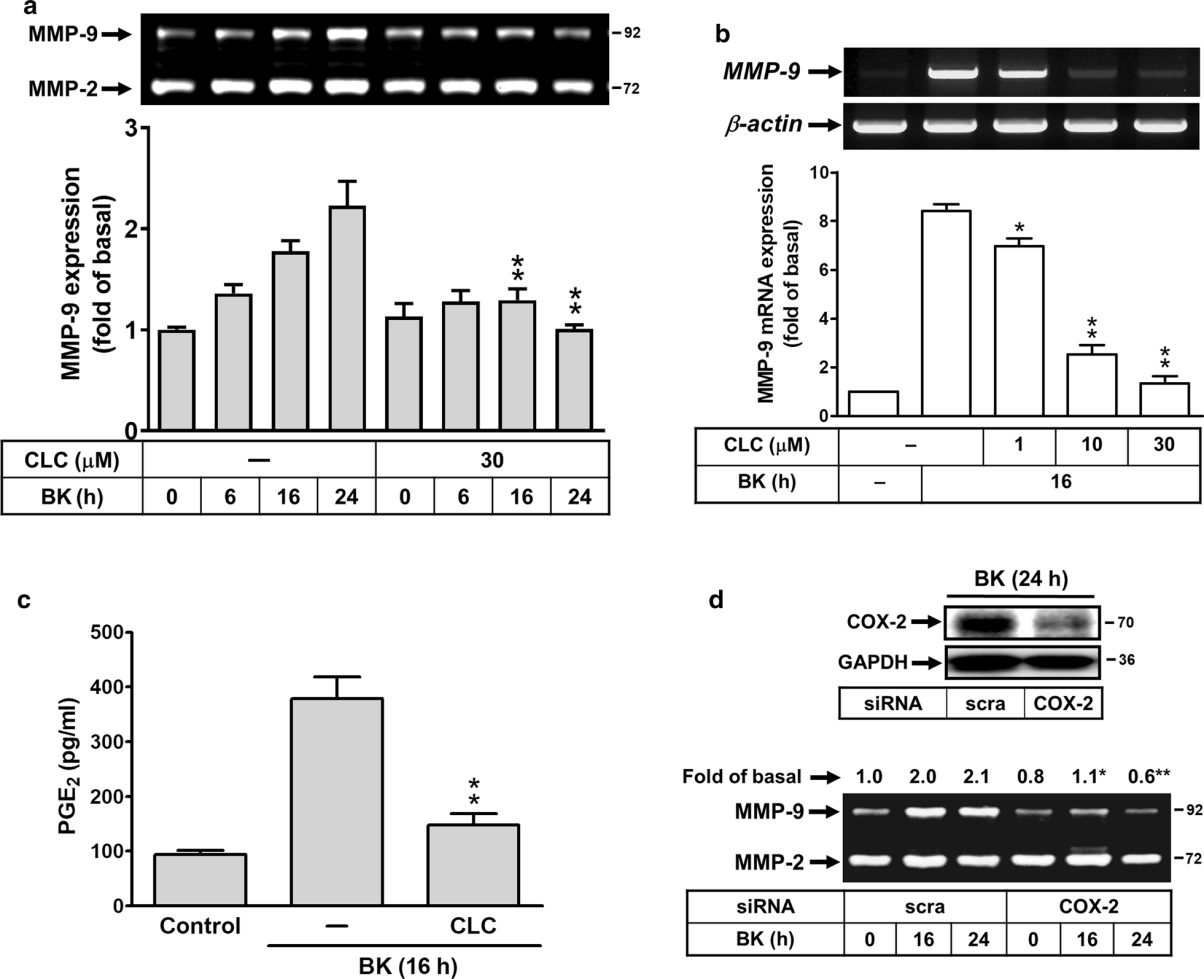
Effect of celecoxib (CLC) on BK-induced MMP-9 up-regulation in RBA cells. **a** Cells were treated with or without celecoxib (CLC, 30 μM) for 1 h before exposure to 10 nM BK for the indicated times. After treatment, the conditioned media were collected and analyzed by gelatin zymography. **b** CLC blocked BK-induced MMP-9 mRNA expression in a concentration-dependent manner, cells were pretreated with various concentrations of CLC (1, 10, 30 μM) for 1 h and then stimulated with BK for 16 h. The total RNA was extracted and analyzed by RT-PCR analysis. **c** Cells were pretreated with CLC (30 μM) for 1 h and then incubated with BK for 16 h. After treatment, the conditioned media were collected and analyzed by PGE_2_-ELISA kit. **d** COX-2 is involved in BK-induced MMP-9 expression, cells were transfected with scramble (scra) or COX-2 siRNA and then treated with or without 10 nM BK for the indicated times. The cell lysates were analyzed by Western blot using an antiserum reactive with COX-2 antibody and membranes were stripped and re-probed with total GAPDH as a control (upper panel). The conditioned media were collected and analyzed by gelatin zymography (lower panel). Data are expressed as mean or mean ± SEM (bar graph) of three independent experiments (N = 3). *P < 0.05; **P < 0.01, as compared with the respective values of cells stimulated with BK only at the same time. The image represents one of three similar experiments

The COX-2/PGE_2_ system is also critical to brain inflammatory diseases [31]. First, we investigate the effect of COX-2/PGE_2_ system on BK-induced MMP-9 expression, rat brain astrocytes (RBA) were pretreated with or without a selective inhibitor of COX-2 activity celecoxib (CLC) for 1 h and then incubated with BK for the indicated time intervals. As shown in Fig. 1a, pretreatment with CLC (30 μM) significantly attenuated BK-induced MMP-9 expression determined by zymography. The result suggested that COX-2 might play a regulatory role in BK-induced MMP-9 expression. We further determined whether COX-2 contributes to BK-induced MMP-9 expression via regulating the transcriptional level, analyzed by RT-PCR. The data showed that pretreatment of RBA with different concentrations of CLC (1, 10, and 30 μM) markedly blocked BK-induced MMP-9 mRNA expression in a concentration-dependent manner (Fig. 1b). These results suggested that COX-2 may be a critical element in BK-induced MMP-9 expression in RBA cells. To further confirm the suggestion, we determined whether BK stimulates the downstream product of COX-2, prostaglandin E_2_ (PGE_2_), increase and the effect of CLC on the event, the conditioned media were collected and measured PGE_2_ levels using an EIA kit. The data showed that BK-induced PGE_2_ biosynthesis was inhibited by pretreatment of cells with CLC (Fig. 1c). Moreover, we found that BK-induced MMP-9 expression was attenuated by knockdown of COX-2 by transfection of RBA cells with the COX-2 siRNA (Fig. 1d). These results demonstrated that COX-2-derived PGE_2_ production may contribute to BK-induced MMP-9 expression in RBA cells.

### BK induces MMP-9 expression via PGE_2_ receptors

**Fig. 2:**
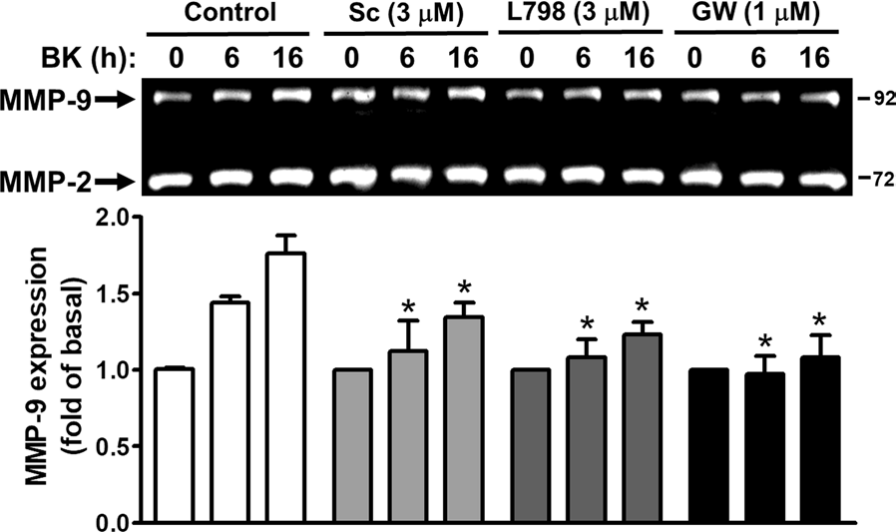
BK induces MMP-9 expression via EP receptors in RBA cells. Cells were pretreated with or without Sc-19220 (Sc, 3 μM), L798-106 (L798, 3 μM), or GW627368 (GW, 1 μM) for 1 h, and then treated with BK (10 nM) for the indicated times. The conditioned media were collected and analyzed by gelatin zymography described under Methods. Data are expressed as mean ± SEM of three independent experiments (N = 3). *P < 0.05, as compared with the respective values of cells stimulated with BK only (control) at the same time. The image represents one of three similar experiments

Next, to determine whether PGE_2_ receptors (E-prostanoid; EP) contribute to BK-induced MMP-9 expression, the EP receptor antagonists were used. The RBA cells were pretreated with the antagonist of EP1 (Sc-19220), EP3 (L798-106), or EP4 (GW627368) and then incubated with BK (10 nM) for the indicated time intervals. The data showed that pretreatment with Sc-19220 (Sc, 3 μM), L798-106 (L789, 3 μM), or GW627368 (GW, 1 μM) attenuated BK-induced MMP-9 expression during the period of observation (Fig. 2), suggesting that BK induces MMP-9 expression via the PGE_2_-dependent EP receptors (*e.g.,* EP1, EP3, and EP4) in RBA cells. These data indicated that BK-induced MMP-9 expression may be mediated through COX-2-derived PGE_2_ autocrine in RBA cells.

### PGE_2_ induces de novo MMP-9 expression via EP receptors

**Fig. 3:**
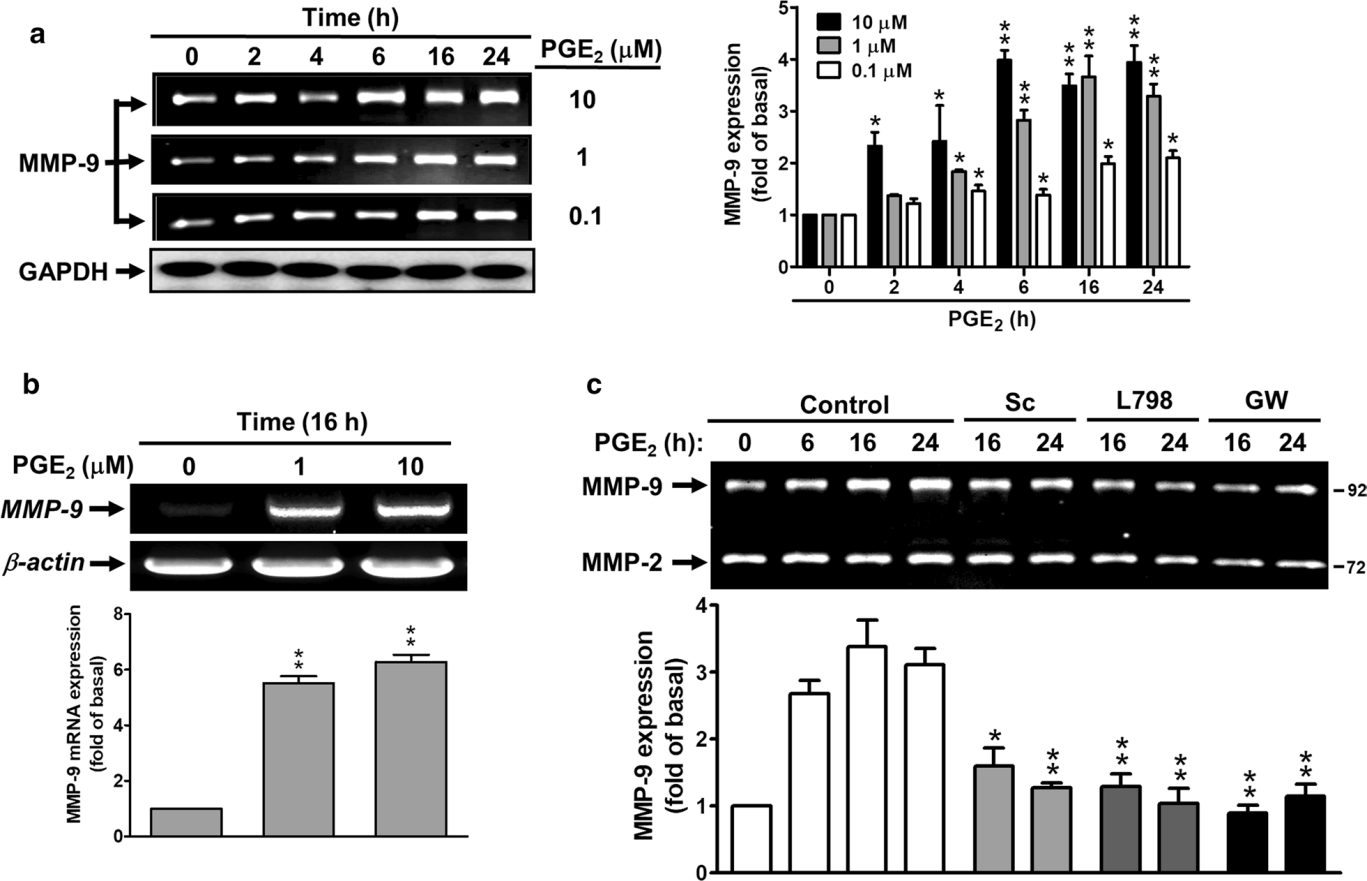
PGE_2_ induces MMP-9 expression via EP receptors in RBA cells. **a** Time and concentration dependence of PGE_2_-induced MMP-9 expression, cells were treated with various concentrations of PGE_2_ (0.1, 1, 10 μM) for the indicated times. After treatment, the conditioned media and cell lysates were collected and analyzed by gelatin zymography (MMP-9) and Western blotting (GAPDH) as described under Methods. **b** Concentration dependence of PGE_2_-induced MMP-9 mRNA expression, cells were treated with PGE_2_ (0, 1, and 10 μM) for 16 h. After treatment, the total RNA were extracted and analyzed by RT-PCR as described under Methods. **c** Cells were pretreated with or without Sc-19220 (Sc, 3 μM), L798-106 (L798, 3 μM), or GW627368 (GW, 1 μM) for 1 h, and then treated with PGE_2_ (10 μM) for the indicated times. The conditioned media were collected and analyzed by gelatin zymography. Data are expressed as mean ± SEM of three independent experiments (N = 3). *P < 0.05; **P < 0.01, as compared with the respective values of cells stimulated with vehicle (**a**, **b**) and PGE_2_ (**c**) only at the same time. The image represents one of three similar experiments

Here, to further demonstrate whether BK-induced PGE_2_ production is important for MMP-9 expression, the RBA cells were directly incubated with PGE_2_ for the indicated time intervals and concentrations. As shown in Fig. 3a, PGE_2_ induced MMP-9 expression in a time- and concentration-dependent manner, a significant increase within 4–24 h. Moreover, we also demonstrated that PGE_2_ induced concentration-dependently MMP-9 mRNA expression by RT-PCR analysis (Fig. 3b). To determine whether PGE_2_-induced MMP-9 expression is mediated through EP receptors, cells were pretreated with the antagonist of EP1 (Sc), EP3 (L798), or EP4 (GW) and then incubated with PGE_2_ (10 μM) for the indicated time intervals. The results showed that pretreatment with Sc (3 μM), L798 (3 μM), or GW (1 μM) suppressed PGE_2_-induced MMP-9 expression during the period of observation (Fig. 3c), indicating that PGE_2_ could indeed induce de novo MMP-9 expression through the EP receptors, including EP1, EP3, and EP4 in these cells.

### Involvement of c-Src in BK- and PGE_2_-induced MMP-9 expression

**Fig. 4:**
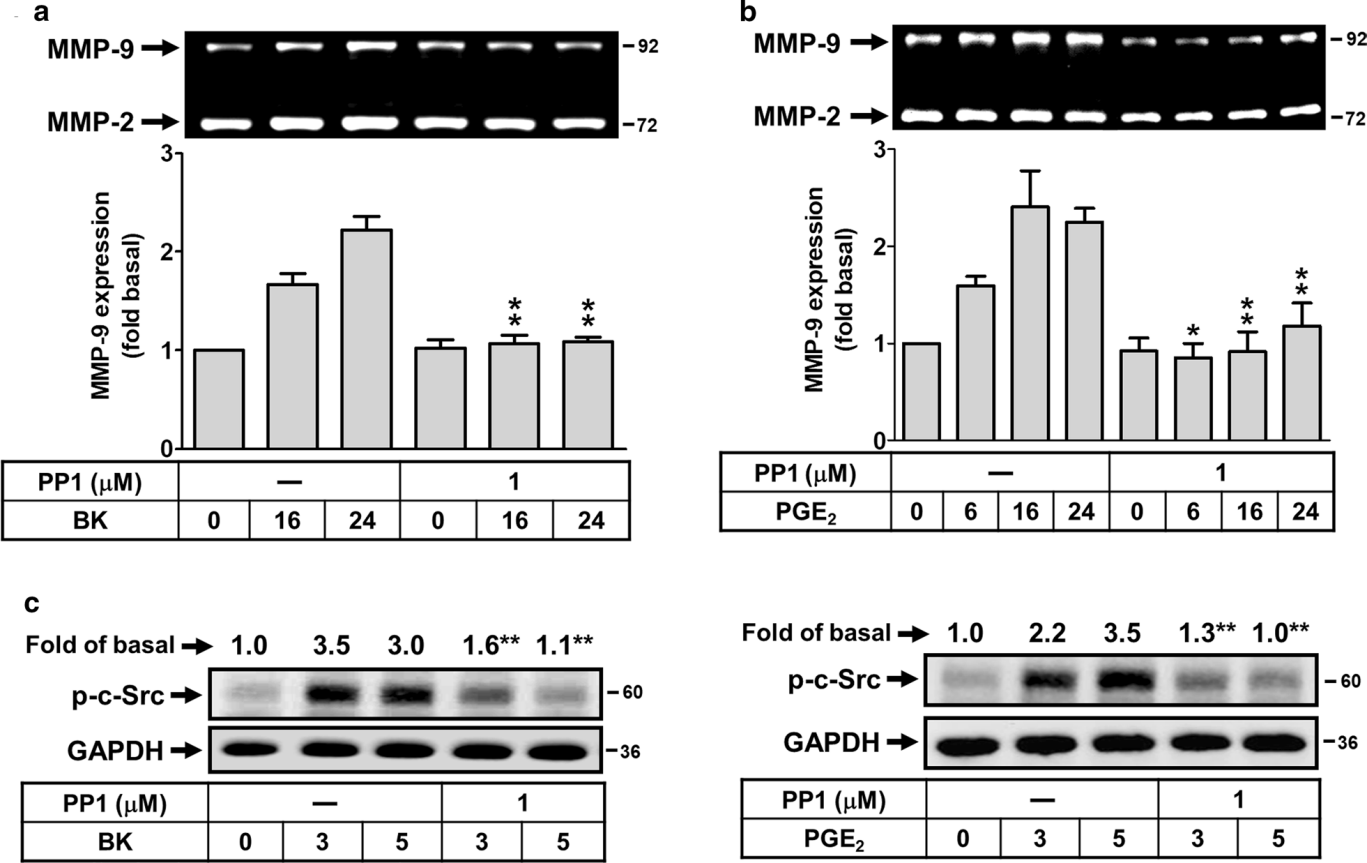
BK induces MMP-9 expression via PGE_2_-stimulated c-Src-dependent manner in RBA cells. **a**, **b** Cells were treated with or without PP1 (1 μM) for 1 h before exposure to 10 nM BK (**a**) or 10 μM PGE_2_ (**b**) for the indicated times. After treatment, the conditioned media were collected and analyzed by gelatin zymorgraphy. **c**, **d** Cells were pretreated with or without PP1 (1 μM) for 1 h and then stimulated with BK (**c**) or PGE_2_ (**d**) for 3 and 5 min. The cell lysates were analyzed by Western blot using an antiserum reactive with phospho-c-Src (p–c-Src) antibody and membranes were stripped and re-probed with total GAPDH as a control. Data are expressed as mean ± SEM (**a**, **b** bar graph) or mean (**c**, **d**) of three independent experiments (N = 3). *P < 0.05; **P < 0.01, as compared with the respective values of cells stimulated with BK (**a**, **c**) or PGE_2_ (**b**, **d**) only at the same time. The image represents one of three similar experiments

To simultaneously investigate the signaling mechanism of BK- and PGE_2_-induced MMP-9 expression, the pharmacological inhibitors of signaling molecules were used. First, we determined the role of c-Src in BK- and PGE_2_-induced MMP-9 expression, cells were pretreated with the inhibitor of c-Src (PP1) for 1 h and then incubated with BK or PGE_2_ for the indicated times. As shown in Fig. 4a, b, pretreatment with PP1 (1 μM) significantly attenuated BK- and PGE_2_-induced MMP-9 expression, suggesting that c-Src was involved in these responses. To further demonstrate the effect of PP1 on BK- and PGE_2_-stimulated c-Src phosphorylation, the phosphorylation of c-Src was analyzed by Western blot. The data showed that pretreatment with PP1 blocked BK-stimulated phosphorylation of c-Src (Fig. 4c, left panel). Additionally, PGE_2_ also stimulate time-dependently c-Src phosphorylation which was blocked by pretreatment of RBA with PP1 (Fig. 4c, right panel). These data suggested that BK induces MMP-9 expression via a PGE_2_-mediated c-Src phosphorylation cascade in these cells.

### PGE_2_ induces MMP-9 expression through EP receptor-mediated ERK1/2 activation

**Fig. 5:**
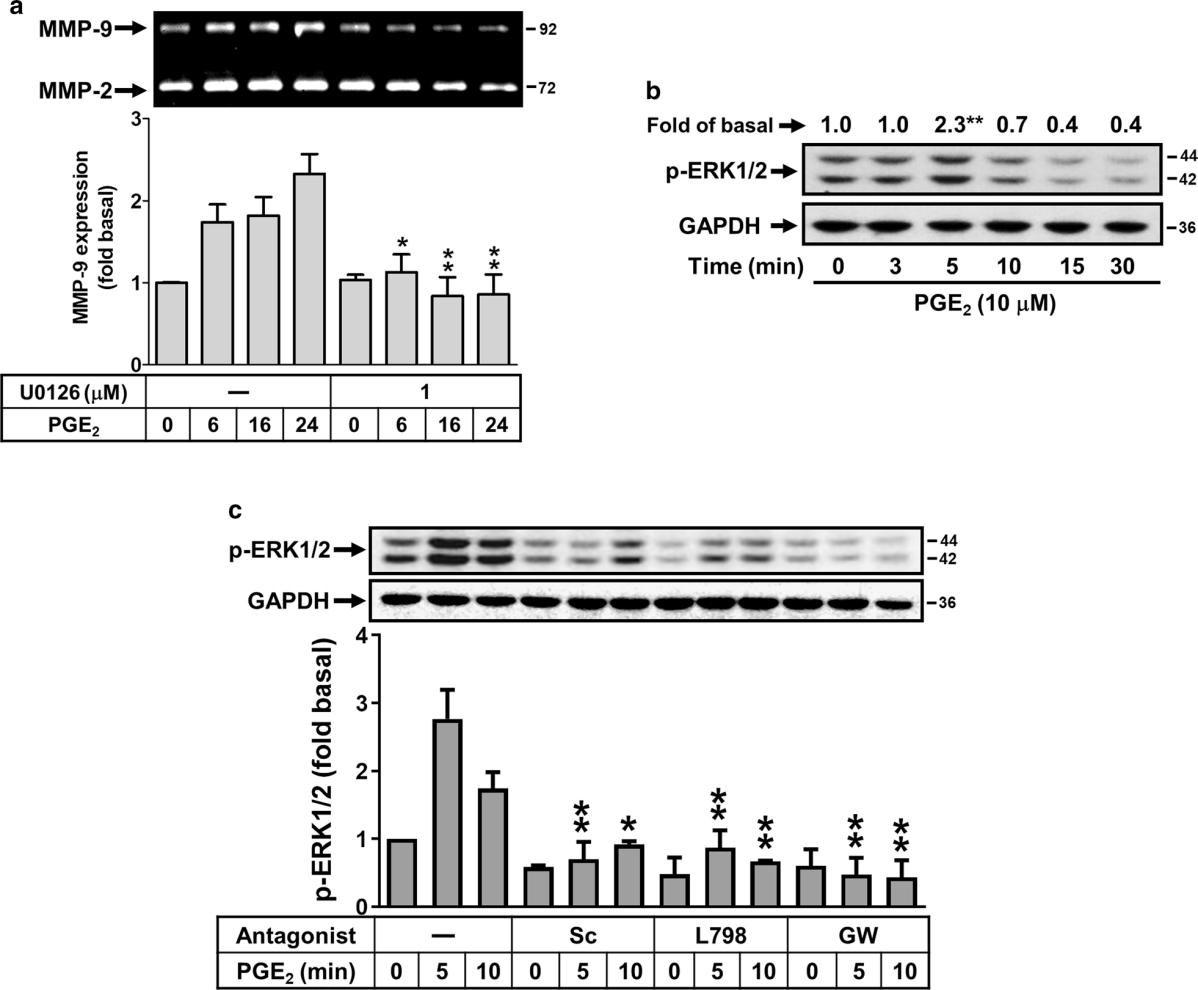
ERK1/2 is involved in PGE_2_-induced MMP-9 expression. **a** Cells were treated with or without U0126 (1 μM) for 1 h before exposure to 10 μM PGE_2_ for the indicated times. After treatment, the conditioned media were collected and analyzed by gelatin zymorgraphy. **b** Cells were stimulated with PGE_2_ (10 μM) for the indicated times and the cell lysates were analyzed by Western blot using an antiserum reactive with phospho-ERK1/2 (p-ERK1/2) antibody and membranes were stripped and re-probed with total GAPDH as a control. **c** Cells were pretreated with or without Sc-19220 (Sc, 3 μM), L798-106 (L798, 3 μM), or GW627368 (GW, 1 μM) for 1 h, and then treated with PGE_2_ (10 μM) for the indicated times. The conditioned media were collected and analyzed by gelatin zymography. Data are expressed as mean ± SEM (**a**, **c**) or mean (**b**) of three independent experiments (N = 3). *P < 0.05; **P < 0.01, as compared with the respective values of cells stimulated with vehicle (**b**) or PGE_2_ (**a**, **c**) only at the same time. The image represents one of three similar experiments

Activation of MAPKs by BK could modulate cellular functions of brain cells [22]. Moreover, the ERK1/2 is involved in BK-induced MMP-9 expression in brain astrocytes [21]. Thus, to determine whether ERK1/2 also participated in PGE_2_-induced MMP-9 expression, cells were pretreated with or without U0126 (1 μM) for 1 h and then incubated with PGE_2_ for the indicated time intervals. As shown in Fig. 5a, PGE_2_-induced MMP-9 expression was attenuated by pretreatment with U0126, suggesting that ERK1/2 may be involved in PGE_2_-induced MMP-9 expression. We further demonstrated that PGE_2_ stimulated time-dependently ERK1/2 phosphorylation by Western blot (Fig. 5b). These results suggested that PGE_2_-induced MMP-9 expression is mediated through ERK1/2 pathway in RBA cells. Next, to determine whether PGE_2_-stimulated ERK1/2 phosphorylation is mediated through EP receptor-dependent pathway, cells were pretreated with the antagonist of EP1 (Sc), EP3 (L798), or EP4 (GW) and then incubated with PGE_2_ (10 μM) for the indicated time intervals. The results showed that pretreatment with Sc (3 μM), L798 (3 μM), or GW (1 μM) significantly blocked PGE_2_-stimulated ERK1/2 phosphorylation during the period of observation (Fig. 5c), suggesting that PGE_2_ stimulated EP receptor (e.g., EP1, EP3, and EP4)-dependent ERK1/2 phosphorylation in these cells. These results demonstrated that PGE_2_-induces MMP-9 expression is mediated through EP receptor-dependent ERK1/2 activation in RBA-1 cells.

### Jak2/STAT3 cascade is required for BK-induced PGE_2_ autocrine linking to MMP-9 expression

**Fig. 6:**
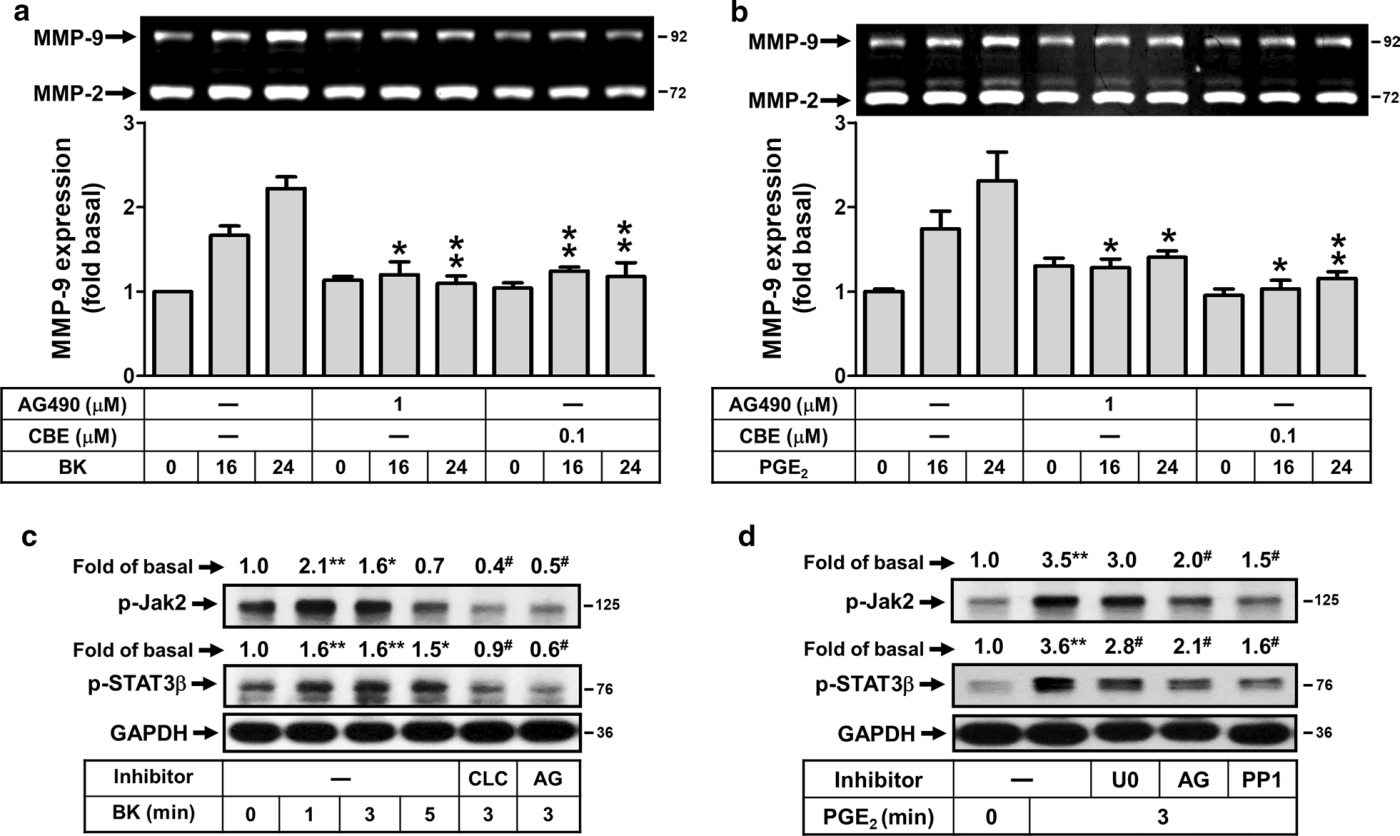
The Jak2/STAT3 cascade participates in BK-induced PGE_2_-dependent MMP-9 expression in RBA cells. **a**, **b** Cells were treated with or without AG490 (1 μM) or CBE (0.1 μM) for 1 h before exposure to 10 nM BK (**a**) or 10 μM PGE_2_ (**b**) for the indicated times. After treatment, the conditioned media were collected and analyzed by gelatin zymorgraphy. **c** Time dependence of BK-stimulated Jak2 and STAT3 phosphorylation, cells were treated with 10 nM BK for the indicated times. Moreover, cells were pretreated with CLC (30 μM) or AG490 (AG, 1 μM) for 1 h and then exposure to 10 nM BK for 3 min. **d** Cells were pretreated with U0126 (U0, 1 μM), AG490 (AG, 1 μM), or PP1 (1 μM) for 1 h and then exposure to PGE_2_ (10 μM) for 3 min. After treatment, the cell lysates were collected and analyzed by Western blotting with anti-phospho-Jak2, anti-phospho-STAT3 or anti-GAPDH as described under Methods. Data are expressed as mean ± SEM (**a**, **b**) or mean (**c**, **d**) of three independent experiments (N = 3). *P < 0.05; **P < 0.01, as compared with the respective values of cells stimulated with BK (**a**) or PGE_2_ (**b**) only at the same time, or basal control (**c**, **d**). ^#^*P* < 0.05, as compared with the respective values of cells stimulated with BK or PGE_2_ only (**c**, **d**). The image represents one of three similar experiments

The Jak/STAT3 cascade is activated upon ligand binding to certain G protein-coupled receptors (GPCRs) including the BK in various cell types like endothelial cells [32]. Moreover, activation of Jak/STAT3 signaling pathway has been shown to regulate MMP-9 expression in tumor invasion and metastasis [33–35]. To examine whether the Jak/STAT3 signaling pathway is also involved in BK-induced COX-2/PGE_2_-mediated MMP-9 expression, the inhibitors of Jak2 (AG490) and STAT3 (CBE: cucurbitacin E) were used. As shown in Fig. 6a, cells were pretreated with AG490 (1 μM) or CBE (0.1 μM) and then incubated with BK (10 nM) or PGE_2_ (10 μM) for the indicated time intervals. The data showed that pretreatment with AG490 (1 μM) or CBE (0.1 μM) both markedly attenuated BK-induced MMP-9 expression. Similarly, pretreatment of cells with AG490 or CBE both also significantly attenuated PGE_2_-induced MMP-9 expression (Fig. 6b). These results suggested that the Jak2/STAT3 cascade was involved in BK- or PGE_2_-induced MMP-9 expression in RBA cells. To further determine whether activation of Jak2/STAT3 cascade in BK-induced responses mediated through phosphorylation of Jak2/STAT3 cascade, as shown in Fig. 6c, BK time-dependently stimulated phosphorylation of Jak2/STAT3 cascade determined by Western blot. A significant response was obtained within 1–3 min. Moreover, pretreatment with the inhibitor of Jak2 (AG) significantly inhibited BK-stimulated phosphorylation of Jak2/STAT3 cascade. We further demonstrate the role of COX-2 in BK-stimulated phosphorylation of Jak2/STAT3 cascade, cells were pretreated with CLC and then incubated with BK for 3 min. The data showed that pretreatment with CLC (30 μM) significantly blocked BK-stimulated phosphorylation of Jak2/STAT3 cascade (Fig. 6c), suggesting that BK-stimulated phosphorylation of Jak2/STAT3 cascade is mediated through COX-2/PGE_2_ system. Subsequently, to confirm the role of COX-2/PGE_2_ system in activation of Jak2/STAT3 cascade, cells were directly incubated with PGE_2_. As shown in Fig. 6d, PGE_2_ stimulated phosphorylation of Jak2/STAT3 cascade at 3 min determined by Western blot. Pretreatment with AG also significantly blocked this PGE_2_ response. To demonstrate the effect of the signaling molecules, including ERK1/2 and c-Src in PGE_2_-stimulated phosphorylation of Jak2/STAT3 cascade, cells were pretreated with U0126 or PP1 and then incubated with PGE_2_ for 3 min. The data showed that pretreatment with PP1 markedly blocked PGE_2_-stimulated phosphorylation of Jak2 and STAT3. Moreover, pretreatment with U0126 inhibited STAT3 phosphorylation, but not Jak2, indicating that PGE_2_-stimulated STAT3 phosphorylation was mediated through c-Src/Jak2/ERK1/2 pathway. The results suggested that BK-stimulated activation of Jak2/STAT3 cascade via COX-2/PGE_2_ system is required for MMP-9 up-regulation in RBA cells.

### Effect of COX-2/PGE_2_ system on BK-induced MMP-9-dependent astrocytic function changes

**Fig. 7:**
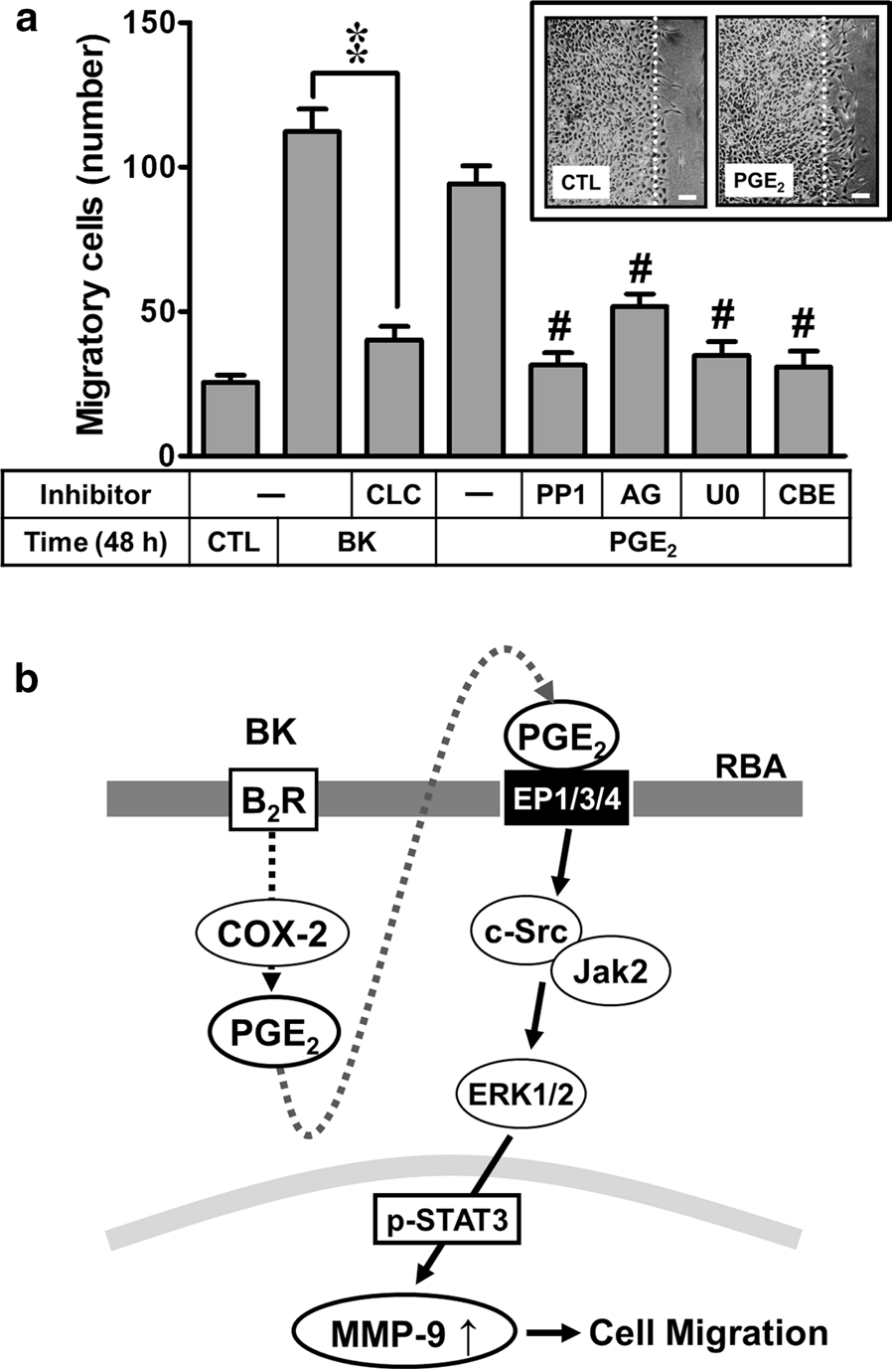
BK induced astrocytic migration through COX-2/PGE_2_-mediated MMP-9 expression pathway in RBA cells. **a** RBA cells were plated on coverslips and grew to confluence, transferred the coverslips to a new 10-cm dish containing serum-free medium for 24 h. Cells were pretreated with CLC (30 μM) for 1 h and then incubated with BK (10 nM) for 48 h. Moreover, cells were pretreated with AG490 (AG, 1 μM), PP1 (1 μM), U0126 (U0, 1 μM, or CBE (0.1 μM) for 1 h and then incubated with PGE_2_ (10 μM) for 48 h. Phase contrast images of RBA cells were taken at 48 h in response to BK or PGE_2_, respectively. Representative images are shown for 48 h (insert panel, scale bar = 20 μm, N = 3). The number of BK- or PGE_2_-induced cell migration at 48 h was counted as described in the Methods. Data are expressed as mean ± SEM of three independent experiments (N = 3). **P < 0.01, as compared with the respective values of cells stimulated with BK only; ^#^*P* < 0.01, as compared with the respective values of cells stimulated with PGE_2_ only. The figure represents one of three similar experiments. **b** Schematic presentation of the role of COX-2/PGE_2_ system in the BK-induced MMP-9 expression and cell migration. In brain astrocytes (RBA), BK induces COX-2/PGE_2_-dependent MMP-9 expression via EP-mediated c-Src, Jak2, and ERK1/2 signals resulting in activation of STAT3. The COX-2/PGE_2_-meditaed MMP-9 expression by BK leads to RBA cell migration

Ultimately, to demonstrate the effect of COX-2/PGE_2_ system on BK-induced MMP-9-dependent astrocytic function changes, we evaluated the cell migration of RBA cells. The images of cell migration induced by BK (10 nM) were observed and taken at 48 h and the number of migratory cells was counted and the statistical data were presented in Fig. 7a. The data showed that pretreatment with CLC (30 μM) significantly blocked BK-induced cell migration, suggesting that the COX-2/PGE_2_ system may be involved in BK-induced astrocytic migration. Moreover, cells were directly incubated with PGE_2_ (30 μM) for 48 h and the images of cell migration were observed and taken at 48 h (Fig. 7a, insert panel). The data showed that pretreatment with PP1 (1 μM), AG (1 μM), U0 (1 μM), or CBE (0.1 μM) all significantly reduced PGE_2_-induced cell migration, suggesting that PGE_2_ could induce astrocytic migration via c-Src/Jak2-ERK1/2-STAT3 cascade. These results demonstrated that COX-2-derived PGE_2_ participated in BK-induced astrocytic migration through activation of c-Src/Jak2-ERK1/2-STAT3 pathway.

## Discussion

Among MMPs, MMP-9 expression and activation play a critical role in tissue remodeling in the pathogenesis of brain diseases [10]. The MMP-9 contributes to a wide range of biological activities in the CNS diseases, including stroke, Alzheimer’s disease, and malignant glioma [10]. Reduction of MMP activity by pharmacological inhibitors or gene knock-out strategies protects the brain from advanced neuroinflammation [36]. These studies suggest that up-regulation of MMP-9 by pro-inflammatory factors may be a great effect upon brain inflammation and neurodegeneration. Moreover, BK and related peptides are simultaneously produced and released following brain injury [37]. Our previous data have demonstrated that BK induces MMP-9 expression in astrocytes which may change astrocytic functions such as cell motility and neuroinflammation [21, 29]. Moreover, BK also induces COX-2 expression in astrocytes [7]. These findings imply that BK may play an important role in brain injury, astroglioma, or the CNS diseases. Pharmacological and knockout-mouse approaches suggest that targeting COX-2 or MMP-9 and their upstream signaling pathways should yield useful therapeutic targets for brain injury and inflammation. Herein, we investigate the effect of COX-2/PGE_2_ system on BK-induced MMP-9 expression in brain astrocytes and its mechanism. In this study, we found that COX-2/PGE_2_ system may be a novel regulator to participate in BK-induced MMP-9 expression in rat brain astrocytes. The results suggest that in brain astrocytes, BK stimulated COX-2-derived PGE_2_ autocrine and further induced MMP-9-dependent astrocytic migration. It is mediated through PGE_2_ receptors (EPs)-linked to the protein kinases (e.g., c-Src and Jak2)-activated ERK1/2 signal leading to induction of STAT3 pathways.

First, we found that a selective COX-2 inhibitor celecoxib (CLC) and knockdown of COX-2 by transfection with siRNA for COX-2 can inhibit BK-induced MMP-9 expression in RBA cells (Fig. 1). A close correlation was observed between the expression of COX-2 under BK-induced conditions and the expression of MMP-9. This result is the first finding that COX-2 can contribute to MMP-9 up-regulation by BK in brain astrocytes. Next, several reports have indicated that COX-2-derived PGE_2_ may up-regulate MMP-9 expression in pancreatic cancer or macrophages [38, 39]. Moreover, a study showed that EP3 receptor signaling on endothelial cells is essential for the MMP-9 upregulation that enhances tumor metastasis and angiogenesis [40]. Thus, we investigated whether BK-induced MMP-9 expression in mediated through PGE_2_ receptors (EPs) in brain astrocytes. The results showed that pretreatment with the antagonist of EP1 (Sc-19220), EP3 (L798-106), or EP4 (GW627368) attenuated BK-induced MMP-9 expression during the period of observation (Fig. 2), suggesting that BK induces MMP-9 expression via the PGE_2_-dependent EP receptors (e.g*.,* EP1, EP3, and EP4) in RBA cells. These data suggested that BK-induced MMP-9 expression may be mediated through COX-2-derived PGE_2_ autocrine in RBA cells.

Accordingly, we presumed that the COX-2-derived PGE_2_ production may contribute to the BK-induced MMP-9 expression in RBA cells. To confirm the hypothesis, the cells were directly stimulated with PGE_2_ (a metabolic product of COX-2). As expected, the data showed that PGE_2_ induced MMP-9 expression in a time- and concentration-dependent manner (Fig. 3a). Moreover, PGE_2_ also induced MMP-9 mRNA expression in RBA cells (Fig. 3b), indicating that COX-2-derived PGE_2_ is involved in BK-induced MMP-9 expression. We further demonstrated that PGE_2_-induced MMP-9 expression via PGE_2_ receptor (EP)-dependent pathways, The results showed that PGE_2_-induced MMP-9 expression was markedly attenuated by pretreatment with various EP antagonists, including EP1, EP3, and EP4 (Fig. 3c), suggesting that PGE_2_-induced MMP-9 expression is mediated through EP (i.e., EP1, EP3, and EP4)-dependent manner in RBA cells. These results demonstrate that an autocrine mechanism of the brain inflammatory responses through cooperation between BK and PGE_2_ to form a positive loop mediating the native COX-2/PGE_2_ production and de novo MMP-9 expression. It is consistent with PGE_2_-induced metalloproteinase 9 (MMP-9) expression and activity occurs through EP-1/EP-3/EP-4 in in cultured monocytic cells [41] and mice lacking COX-2 or EP4 in bone marrow-derived cells show a reduced expression of MMP9, which results in decreased infiltration of monocytes and T cells into the CNS [42].

Many reports and our previous data have indicated that several protein kinases such as c-Src may contribute to various stimuli-induced MMP-9 expression in several cell types [43–45]. Moreover, several reports also demonstrate that c-Src is crucial for MMP-9 expression in brain astrocytes [46, 47]. Here, the data showed that BK induced the expression of MMP-9 was attenuated by PP1 (Fig. 4a). Similarly, pretreatment with PP1 significantly inhibited PGE_2_-induced MMP-9 expression in RBA cells (Fig. 4b). Moreover, BK or PGE_2_ can stimulate phosphorylation of c-Src which was significantly blocked by PP1 (Fig. 4c), indicating that c-Src phosphorylation plays an important role in PGE_2_-induced MMP-9 expression, consistent with BK-induced MMP-9 expression through c-Src revealed by zymography in RBA cells. These results are consistent with up-regulation of MMP-9 by c-Src in IL-1β induction in brain astrocytes [43], in TNF-α stimulation in osteoblast-like MC3T3-E1 cells [44], and in thrombin-induced neuroblastoma SK-N-SH cell migration [45].

Herein, we further investigated the involvement of MAPKs in PGE_2_-induced MMP-9 using a specific pharmacological MAPK inhibitor, U0126, SB202190, and SP600125. The expression of MMP-9 by PGE_2_ was markedly inhibited by U0126, but not SB202190 and SP600125 (Fig. 5a), suggesting that PGE_2_-induced MMP-9 expression is mediated through an ERK1/2-dependent mechanism. As expected, we found that PGE_2_ stimulated ERK1/2 phosphorylation in a time-dependent manner (Fig. 5b). Pretreatment with antagonist of EP1 (Sc-19220), EP3 (L798-106), and EP4 (GW627368) all significantly inhibited PGE_2_-stimulated ERK1/2 phosphorylation (Fig. 5c). These results suggest that ERK1/2 MAPK participate in PGE_2_-induced MMP-9 expression in RBA cells. Moreover, our previous reports have shown that BK induces MMP-9 expression via c-Src-dependent ERK1/2 pathway [46]. These results suggested that BK-induced MMP-9 expression is mediated through PGE_2_-dependent EP(1/3/4) linking to c-Src/ERK pathway in RBA cells, consistent with PGE_2_-induced MMP-9 expression in dendritic cells through activation of ERK [48] and up-regulation of cPLA_2_ by BK through EP-mediated ERK1/2 activation in brain astrocytes [30].

Janus kinases (Jaks) are a family of four tyrosine kinases (Jak1, Jak2, Jak3 and Tyk2) that selectively associate with cytokine receptor chains and mediate signaling by phosphorylating tyrosine residues on various proteins in the pathway, including STAT (signal transducer and activator of transcription) transcription factors [49–51]. The Jak/STAT signaling pathway is implicated in the pathogenesis of inflammatory, autoimmune, and degenerative diseases including rheumatoid arthritis [52]. In the CNS, Jak/STAT cascade is a critical part of several intracellular signaling events that regulate many pathophysiological functions. A report has indicated that age- and disease-dependent deterioration in the Jak2/STAT3 axis plays a critical role in the pathogenesis of Alzheimer's disease [53]. These studies suggest that Jak/STAT may play a critical role in regulation of inducible gene expression in inflammatory responses. Therefore, we further investigated the role of Jak/STAT pathway in BK- or PGE_2_-induced MMP-9 expression in brain astrocytes. The results showed that pretreatment with AG490 (a Jak2 inhibitor) and CBE (a STAT3 inhibitor) both significantly blocked BK-induced MMP-9 expression (Fig. 6a), indicating that Jak2 and STAT3 are involved in BK-induced MMP-9 expression. The result is consistent with promotion of cell migration and invasion MMP-9 through the Jak2/Stat3/MMP9 signaling pathway in B7‑H3 stimulation in colorectal cancer [54]. Moreover, the data showed that BK can stimulate phosphorylation of Jak2 and STAT3β in a time-dependent manner which were attenuated by pretreatment with a selective COX-2 inhibitor celecoxib (CLC) and AG490 (Fig. 6c), suggested that BK-stimulated phosphorylation of Jak2 and STAT3β are mediated through COX-2-dependent pathway. The results also indicated that BK stimulates STAT3β phosphorylation via Jak2-mediated manner. We further demonstrated whether BK induces MMP-9 expression via COX-2/PGE_2_-dependent activation of Jak2/STAT3 pathways, RBA cells were directly treated with PGE_2_. Predictably, PGE_2_-induced MMP-9 expression was markedly attenuated by pretreatment with AG490 and CBE (Fig. 6b), indicating that Jak2 and STAT3 are involved in the response. Similarly, the data showed that PGE_2_-stimulate phosphorylation of Jak2 and STAT3β were attenuated by pretreatment with AG490 (Fig. 6d), indicated that PGE_2_-stimulated phosphorylation of STAT3β is mediated through Jak2-dependent pathway.

Moreover, previous reports have indicated that the best characterized interactions of the Jak/STAT pathway are with the MAPKs [49]. The MAPKs specifically phosphorylates a serine near the C terminus of most STATs that will enhance transcriptional activation by STAT [49]. Thus, the MAPKs (*i.e.* ERK1/2, JNK1/2, and p38 MAPK) are key signaling enzymes that couple receptor activation to gene transcription by phosphorylating STATs. Our data showed that PGE_2_-induced STAT3β phosphorylation, but not Jak2, was attenuated by U0126, suggesting that phosphorylation of STAT3β is mediated through ERK1/2 pathway (Fig. 6d). Moreover, pretreatment with PP1 also attenuated PGE_2_-stimulated Jak2 and STAT3β phosphorylation (Fig. 6d), indicating that c-Src may be an upstream regulator of Jak2/STAT3β cascade in RBA cells. These results suggested that COX-2/PGE_2_ system-dependent activation of Jak2/STAT3β cascade is a novel and critical pathway for BK-induced MMP-9 expression in brain astrocytes. Moreover, PGE_2_-stimulated STAT3β phosphorylation is mediated through c-Src/Jak2 linking to phosphorylation of ERK1/2 in these cells. For the role of STAT3, we are the first presented that STAT3β plays a critical role in induction of MMP-9 by BK and PGE_2_ in brain astrocytes (RBA). Taken together, these results suggest key roles of PGE_2_ autocrine and STAT3 in the severity of brain inflammation through up-regulation of MMP-9 in brain astrocytes.

## Conclusions

In summary, we showed that BK induced expression of MMP-9 via COX-2-dependent PGE_2_ production leading to PGE_2_ receptor (EP)-mediated pathways. Subsequently, the autocrine PGE_2_-induced MMP-9 expression is mediated through EP(1/3/4)-dependent c-Src/Jak2/ERK1/2 linking to STAT3 activation in RBA cells. Finally, BK-induced MMP-9-dependent RBA cell migration is also mediated through these pathways. Based on the observations from literatures and our findings, Fig. 7b depicts a model for the molecular mechanisms underlying BK-induced COX-2/PGE_2_-dependent MMP-9 expression and cell migration (Fig. 7a) of RBA cells. These findings concerning BK-induced MMP-9 gene expression through a novel and PGE_2_ autocrine regulation in brain astrocytes imply that BK, COX-2/PGE_2_ system, and MMP-9 play an important role in amplifying brain inflammation and CNS diseases. Pharmacological approaches suggest that targeting COX-2/PGE_2_ system and Jak/STAT cascade signaling components would yield useful therapeutic targets for brain inflammatory diseases.

## Data Availability

The datasets used and/or analyzed during the current study are available from the corresponding author on reasonable request.

## Abbreviations

CNS: Central nervous system
BBB: Blood–brain barrier
BK: Bradykinin
MMP-9: Matrix metalloproteinase-9
COX-2: Cyclooxygenase-2
PGE_2_: Prostaglandin E_2_
RBA: Rat brain astrocytes
STAT3: Signal transducer and activator of transcription 3
DMEM/F-12: Dulbecco’s modified Eagle’s medium/Ham’s nutrient mixture F-12
FBS: Fetal bovine serum
ECL: Enhanced chemiluminescence
BCA: Bicinchoninic acid
PBS: Phosphate-buffered saline
GPCR: G protein-coupled receptor
siRNA: Small interfering RNA
RT-PCR: Reverse transcription-polymerase chain reaction
EIA: Enzyme immunoassays
